# Acceptance and Commitment Therapy and Exercise: Can They Help Older Adults Manage Chronic Pain?

**DOI:** 10.1093/geroni/igab046.1118

**Published:** 2021-12-17

**Authors:** Dara K Y Leung, Annabelle P C Fong, Wai-Wai Kwok, Angie K Y Shum, Tianyin Liu, Gloria H Y Wong, Terry Y S Lum

**Affiliations:** The University of Hong Kong, Hong Kong, Hong Kong

## Abstract

Chronic pain is common among older adults and affects their physical and psychological well-being. While exercise can reduce pain and promote physical functions, psychological interventions may enhance pain management by addressing the psychosocial contributors to the prolonged pain. Acceptance and Commitment Therapy (ACT) is a psychological intervention that emphasizes on psychological flexibility, values, and mindfulness. This approach may be particularly helpful in dealing with chronic pain, where symptoms can be beyond one’s control. This single group pre-post study investigated the feasibility and efficacy of an intervention combining ACT and exercise for chronic pain management in older adults. The intervention consisted of 16 sessions delivered over eight weeks. ACT and exercises were modified according to the individual’s capability when needed. Clinical outcomes regarding pain severity and interference, pain acceptance, value of life, depression, anxiety, and physical functioning were assessed. Twenty-four older adults attended all sessions and completed the assessments. Preliminary results showed that, while participants experienced similar level of pain after the intervention, they reported less pain interference on mood and enjoyment of life, and improved chronic pain acceptance, pain self-efficacy, success at living their values, committed action, depressive symptoms, physical functioning in the lower body strength, aerobic and endurance, agility and dynamic balance, and upper body strength (all p<.050). This study lends support to the feasibility of a combined ACT and exercise intervention for chronic pain management in older adults. The efficacy of ACT may not be directly on reducing pain, but on increased psychological flexibility to co-live with pain.

